# Supplementary material for: Effects of quarantine on Physical Activity prevalence in Italian Adults: a pilot study
Source: PeerJ. 2022 Oct 3;10:e14123. doi: 10.7717/peerj.14123 (PMC9536321; doi:10.7717/peerj.14123)
Supplement: Supplemental Information 2 [file peerj-10-14123-s002.docx]

General information

Please insert the first word of your first name, the first word of your last name and the last two number of your birthdate (example: Mario Rossi, January 1, 1980, becomes MR80)

___________________

Gender:

- Female
- Male

How old are you? (Years old)

_________

Educational Status:

- Primary School
- Secondary School
- High School
- Degree
- Master’s degree
- PhD
- Other

Occupational Status:

- Student
- Employed
- Freelance
- Unemployed
- Homemade
- Retired
- Other

Did you practice PA and/or sports before the COVID-19 quarantine?

- Yes
- No

Did you practice PA and/or sports during the COVID-19 lockdown?

- Yes
- No

If you answered to the previous question “No”, why?

- Covid-19 problems
- Environmental problems
- Health problems
- Psycho-social aspects
- Technological problems

If you answered to previous question “Yes”, why?

- General well-being
- Physical well-being
- Psychological well-being
- Physical and General well-being
- Psychological and General well-being
- Psychological and Physical well-being

Did you practice PA and/or sports after the COVID-19 lockdown?

- Yes
- No

If you answered to the previous question “No”, why?

- Covid-19 problems
- Environmental problems
- Health problems
- Psychological aspects
- Socio-Economics problems

The following questions ask you for information on Physical Activity during the COVID-19 lockdown and on socio-political decisions adopted by the Italian government to contain the virus’s spread. Please select one of the possible answers considering that “1” means you disagree with the concern and “4” means you agree with the concern.

| Questions | Disagree | Partially Disagree | Partially Agree | Agree |
| --- | --- | --- | --- | --- |
| Do you think the lack of practice of physical activity and/or sport during the lockdown has negatively affected your health? | 1 | 2 | 3 | 4 |
| Do you think the lack of practice of physical activity and/or sport in older adults during lockdown has negatively affected their health? | 1 | 2 | 3 | 4 |
| Do you think the lack of practice of physical activity and/or sport during lockdown will cause problems for the national health system and public health? | 1 | 2 | 3 | 4 |
| Do you think the lack of practice of physical activity and/or sport could have affected emotional/psychological wellbeing during the lockdown? | 1 | 2 | 3 | 4 |
| Do you think the lack of practice of physical activity and/or sport could have affected emotional/psychological growth in children/adolescents during the lockdown? | 1 | 2 | 3 | 4 |
| Do you think children/adolescents practised enough amount of physical activity and/or sport during the lockdown? | 1 | 2 | 3 | 4 |
| Do you think the lack of practice of physical activity and/or sport could have affected emotional/psychological status in older adults (>65 years old) during the lockdown? | 1 | 2 | 3 | 4 |
| Do you think the Italian government adequately considered the practice of physical activity and/or sport during the lockdown? | 1 | 2 | 3 | 4 |
| Do you think the usage of tech devices to practice physical activity and/or sport during lockdown could have affected social interaction negatively? | 1 | 2 | 3 | 4 |
| Do you think sports federations and societies adequately promoted the practice of physical activity and/or sport during the lockdown? | 1 | 2 | 3 | 4 |
| Do you think public and private schools adequately promoted the practice of physical activity and/or sport during the lockdown? | 1 | 2 | 3 | 4 |
