# Supplementary material for: Effects of quarantine on Physical Activity prevalence in Italian Adults: a pilot study
Source: PeerJ. 2022 Oct 3;10:e14123. doi: 10.7717/peerj.14123 (PMC9536321; doi:10.7717/peerj.14123)
Supplement: Supplemental Information 3 [file peerj-10-14123-s003.docx]

**Informazioni Generali**

Gentilmente inserisca la prima lettera del suo nome, la prima lettera del suo cognome e i gli ultimi due numeri del suo anno di nascita (esempio: Mario Rossi, 1980, diventa MR80)

___________________

Genere:

- Femminile
- Machile

Età? (anni)

_________

Titolo di studio:

- Licenza scuola primaria
- Licenza scuola secondaria
- Diploma/ licenza liceale
- Laurea triennale
- Laurea magistrale
- Dottorato
- Altro

Occupazione:

- Studente
- Occupato
- Libero professionista
- Inoccupato
- Casalingo
- Pensionato
- Altro

**Attività Fisica**

Praticava regolarmente attività fisica e/o sport prima del Covid-19?

- Si
- No

Praticava regolarmente attività fisica e/o sport durante il lockdown?

- Si
- No

Se ha risposto “No” alla domanda precedente, perché?

- Problemi correlati al Covid-19
- Problemi ambientali
- Problemi di salute
- Aspetti psico-sociali
- Problemi con l’utilizzo della tecnologia

Se ha risposto “Si” alla domanda precedente, perché?

- Benessere generale
- Benessere fisico
- Benessere Psicologico
- Benessere fisico e generale
- Benessere Psicologico e generale
- Benessere Psicologico e fisico

Adesso che il lockdown è terminato, pratica regolarmente attività fisica e/o sport?

- Si
- No

Se ha risposto “No” alla domanda precedente, perché?

- Problemi correlati al Covid-19
- Problemi ambientali
- Problemi di salute
- Aspetti psicologici
- Problemi socio-economici

Le seguenti domande le chiederanno informazioni relative all’attività fisica/sport praticata durante il lockdown di marzo-maggio 2020, e le verrà chiesto di esprimere il suo parere in merito ad alcune decisioni adottate dal governo italiano per contenere la diffusione del virus. Le chiediamo di selezionare solo una delle possibili risposte considerando che “1” indica uno stato di disaccordo con quanto riportato e “4” indica uno stato di accordo con quanto scritto nel testo.

| Questions | Disagree | Partially Disagree | Partially Agree | Agree |
| --- | --- | --- | --- | --- |
| Pensa che l’assenza di attività fisica e/o sport durante il lockdown abbia avuto effetti negativi sulla sua salute? | 1 | 2 | 3 | 4 |
| Pensa che l’assenza di attività fisica e/o sport nelle persone anziane durante il lockdown abbia avuto effetti negativi sul loro stato di salute? | 1 | 2 | 3 | 4 |
| Pensa che l’assenza di attività fisica e/o sport durante il lockdown possa essere una minaccia per il sistema sanitario nazionale e la salute pubblica? | 1 | 2 | 3 | 4 |
| Pensa che l’assenza di attività fisica e/o sport durante il lockdown abbia avuto effetti sul benessere psico-emotivo dei cittadini? | 1 | 2 | 3 | 4 |
| Pensa che l’assenza di attività fisica e/o sport durante il lockdown possa aver avuto effetti negativi sullo sviluppo psico-emotivo dei bambini/ adolescenti? | 1 | 2 | 3 | 4 |
| Ritiene che I bambini/ adolescenti abbiano praticato una sufficiente quantità di attività fisica/ sport durante il lockdown? | 1 | 2 | 3 | 4 |
| Pensa che l’assenza di attività fisica e/o sport durante il lockdown abbia avuto conseguenze negativo sullo stato psico-emotivo degli anziani (>65 anni)? | 1 | 2 | 3 | 4 |
| Ritiene che il governo italiano abbia attribuito il giusto peso alla pratica di attività fisica/ sport durante il lockdown? | 1 | 2 | 3 | 4 |
| Pensa che l’uso di dispositivi tecnologici per la pratica dell’attività fisica e/o sport abbia avuto conseguenze negative sull’interazione sociale? | 1 | 2 | 3 | 4 |
| Ritiene che le federazioni e gli enti sportivi abbiano promosso adeguatamente la pratica di attività fisica e/o sport durante il lockdown? | 1 | 2 | 3 | 4 |
| Ritiene che le scuole private e pubbliche abbiano promosso adeguatamente la pratica di attività fisica e/o sport durante il lockdown? | 1 | 2 | 3 | 4 |
